# The Weather

**Published:** 1846-01

**Authors:** 


### THE WEATHER.

The month of December was the coldest month that has been experienced in this region for several years. The mean of February, 1841, we think it was, was 30°, since which time we have not known so cold a month until the present time. The mean temperature of December was 25°. Having had an early spring, a hot summer, and a late, mild autumn, we were prepared to see a cold winter. As the average of the three preceding seasons had been too high for our latitude, it was pretty certain that the annual mean would be restored by the only remaining season of the year. The latter part of November was intensely cold—one morning the thermometer in Louisville having stood at 4°, and in Jacksonville, Illinois, by report, 26° below zero. The mean of the four last mornings in the month was 16°. The mean temperature of December, at sun rise, was 19.2°; the highest rise at any time during the month was 48°; the lowest point to which it fell was 0°; for three successive mornings the range was 1°, 0°, 8°, and during two successive days the mean was as low as 5°.

December, it will hence appear, with the latter days of November, has gone far towards reducing the excessive heat of the previous seasons to the annual mean, and we may therefore look for open weather in January or February. The river was closed by ice early in December; the prediction of our meteorologists, founded upon the theory of a mean annual temperature, has been, that a thaw would occur, and navigation be resumed, before the end of January.

In connection with the weather, it seems appropriate to say something in regard to the prevailing diseases of the season. These, as was to have been expected, were of an inflammatory type—catarrhs, attended with acute pain in the frontal sinus in some cases, and with tonsilitis, in many, pleurisy and pneumonia. We witnessed a highly interesting case of each of the latter of these affections. The first a case of pleurisy, was presented in a little girl, about 7 years of age. It was complicated, after the first few days, with symptoms of gastritis. The case of pneumonia was a highly aggravated one; the subject of it was a lady, aged about 60 years, corpulent, and of excellent constitution. She was bled freely on the first day of her attack, and put upon calomel and antimony. The second night afterwards the symptoms assumed the most alarming aspect; the dyspnœa was excessive, attended with lividity of countenance, and apoplectic signs. The venesection was repeated, and in the several bleedings amounted to ninety-six ounces of blood. Stimulants became necessary in the progress of the case, the result of which was favorable, as was also that of the case of pleurisy. The city may be said, at the present time, to be very healthy. Y.

